# Vancomycin prescribing and therapeutic drug monitoring: Challenges of real clinical practice

**DOI:** 10.1371/journal.pone.0285717

**Published:** 2023-05-17

**Authors:** Mariam Hantash Abdel Jalil, Rima Ηijazeen, Farah Khaled Abu-Mahfouz, Khawla Abu Hammour, Maria Hasan Matalqah, Jwan Saleh Khaleel Albadaineh, Shrouq Khaled AlOmoush, Montaha Al-Iede

**Affiliations:** 1 Department of Biopharmaceutics and Clinical Pharmacy, The University of Jordan, Amman, Jordan; 2 School of Pharmacy Jordan University of Science and Technology, Irbid, Jordan; 3 School of Pharmacy Mu’tah University, Kerak, Jordan; 4 Department of Pediatrics, Jordan University Hospital, Amman, Jordan; 5 School of Medicine, The University of Jordan, Amman, Jordan; American University of Beirut Medical Center, LEBANON

## Abstract

**Background:**

Vancomycin prescription and monitoring guidelines have been reported to be poorly followed by various centers.

**Aims:**

Identifying barriers to compliance with vancomycin dosing and therapeutic drug monitoring guidelines (TDM) and possible ways to enhance compliance based on the healthcare providers’ (HCPs) perspective.

**Methods:**

A qualitative study based on semi-structured interviews with HCP (physicians, pharmacists, and nurses) was conducted at two Jordanian Teaching Hospitals. Interviews were audio-recorded and analyzed through thematic analysis. The COREQ criteria for qualitative research were utilized to report the study findings.

**Results:**

A total of 34 HCPs were interviewed. HCP perceived several factors as barriers to guideline recommendation compliance. Such factors included negative perception towards prescription guidelines, lack of knowledge regarding TDM guidelines, the hierarchy of medication management, work pressure, and ineffective communication among healthcare providers. Potential strategies to optimize guidelines adaptation included providing HCPs with more training and decision support tools in addition to activating the role of clinical pharmacists.

**Conclusions:**

The main barriers to guideline recommendations uptake were identified. Interventions should address those barriers related to the clinical environment, including enhancing interprofessional communication related to vancomycin prescription and TDM, reducing workload and providing support systems, promoting educational and training programs, in addition to adopting guidelines suitable for the local environment.

## Introduction

Vancomycin, a glycopeptide antibiotic, is of the most frequently used antibiotics, especially in critically ill patients and pediatric wards [1–3]. It is considered the first-line antibiotic for managing serious methicillin-resistant Staphylococcus aureus (MRSA) infections such as sepsis, meningitis, and endocarditis. Unfortunately, vancomycin has been commonly implicated in causing medication related-patient harm due to its narrow therapeutic index [4]. Prescribing antibiotics, in general, can be challenging for prescribers. For instance, underdosing has been frequently described [5, 6]. Furthermore, overuse of antibiotics is prevalent. This might be associated with the fact that physicians lacking enough experience can have the perception that the benefits of antibiotic treatment out ways long-term risks, which include resistance and reduced efficacy [7]. For vancomycin as an example, many researchers reported excessive and prolonged empiric prescription [8, 9]. To guide its use and minimize adverse drug effects due to overdosing and inefficacy due to underdosing, Therapeutic Drug Monitoring (TDM) is recommended for prolonged courses. At the study institutions, vancomycin TDM is based mainly on trough monitoring. Despite its simplicity, compared to other TDM methods, many researchers reported suboptimal trough monitoring practice.

In previous research of ours [10], we measured the appropriateness of vancomycin dosing and monitoring practices at Jordan University (JUH) teaching hospital. This study revealed several defects in the vancomycin clinical practice, which most prominently included prolonged antibiotic treatment, inaccurate documentation of TDM parameters, and poor clinical action in response to markedly low drug levels (< 7 mg/L). Similar results were also reported by other researchers, and several studies evaluated barriers to appropriate dosing and/or monitoring of antibiotics among hospital physicians [7, 11, 12]. However, vancomycin dosing, administration, and TDM require collaborative efforts between physicians, pharmacists, and nurses. For this reason, the present study aimed to explore perceived barriers to appropriate vancomycin prescribing, administration, and TDM practices by all healthcare providers (HCP). Furthermore, it aimed to explore possible strategies to optimize these practices as perceived by the HCP.

## Methodology

### Study design and ethics

The current study was a qualitative study based on face-to-face semi-structured interviews with HCPs involved in vancomycin prescription/ TDM. The study was conducted over a period of two months, starting on August 2021 and extending until October 2021. The study was conducted in two university teaching hospitals in Jordan. The first was the Jordan University Hospital (JUH). JUH is the first university teaching hospital in Amman, the Capital of Jordan, with a capacity of up to 600 beds. The second is the King Abdallah University Teaching Hospital (KAUH). It is considered the largest medical structure in the north of Jordan, with a bed capacity of 678. Institutional Review Board approvals were obtained from participating centers (JUH and KAUH).

### Data collection and interpretation

Residents, pharmacists, and nurses with specific experiences and roles in vancomycin dosing/TDM and were willing to provide a detailed description of their personal experience were eligible to participate, provided that they signed written informed consent.

Consultant physicians were not included in the present study. Based on our previous research on vancomycin prescription and monitoring [10], residents are more involved in daily dosing and monitoring. This approach was also adopted by other researchers [13]. Furthermore, due to their important influence on the prescribing behavior of residents, it was deemed better by the research team to evaluate their opinions in a separate study that includes more study sites.

Initially, eligible HCP were sent an invitation letter to participate in this semi-structured interview. Later, a convenience snowball sampling method was used as HCPs recommended colleagues that could be willing to take part. Participation was voluntary, and no compensation for the time spent in the interview was offered to participants.

HCPs were provided with written information about the study and asked to arrange a location of the HCP’s choice and a mutually convenient time for the interview. The number of interviews was determined by the point where the data was deemed saturated (i.e., no new themes emerged).

An interview guide was developed based on a review of the literature [12, 14] and input from an expert in qualitative research (R.H, Ph.D.) and clinical pharmacokinetics and pharmacy practice (M.A, K. A) (S1 File). The interview guide was written in Arabic language and was formulated as open, non-leading questions and focused on vancomycin prescription practices and associated barriers and challenges faced during usual vancomycin prescribing and TDM practices, in addition to potential means for improving vancomycin prescribing and TDM practice based on HCP views. Prompts and follow-up questions were used as appropriate. The interview guide was pilot-tested prior to finalization (results not included in the analysis). One female clinical pharmacist with previous experience in research (F. K) conducted the interviews at JUH, while senior PharmD students, both females (M.M, S.A), conducted the interviews at KAUH. All of them received training on interviewing and conducted pilot interviews with (R.H.) before starting the study. None of them had an established relationship with the participant before the start of the study.

The Consolidated Criteria for Reporting Qualitative Research (COREQ) criteria for qualitative research were utilized to report the study findings [15]. All interviews were audio-recorded and de-identified. The interviews took place at the workplace during working hours, usually in a meeting room or a physician’s office. The interview schedule began after the interviewer introduced herself as a clinical pharmacist/research assistant. Later a summary of the research rationale and objectives and an overview of the interview guide were provided. Anonymized audio records were transcribed verbatim. The data collected were translated from Arabic to English and then back-translated again to Arabic to assure accuracy. No interviews were repeated, nor did participants hear their records after the interview due to limitations of time. Transcripts were double-checked for accuracy following the transcribing. Data were analyzed using thematic framework analysis [16]. The researchers (F.K, S.K, M.M) kept a record of reflections and thoughts about interpretations of the collected data, which included illustrations of possible relationships between emerging themes to guide or reflect the analysis. Those themes were revised and refined through discussion with all authors to ensure that the analysis was supported by the data. Similar to the approach of Chan *et al*. [12], the data were not represented quantitatively to prevent misrepresentation due to the versatile type of discussions in each semi-structured interview.

## Results

A total of 34 HCPs working at different hospital wards, such as pediatric wards, internal wards, surgical and intensive care units, we interviewed in the present study. Due to the design of the study response rate could not be determined. The interviews ranged in duration from 11 to 38 minutes, and data saturation was achieved. Table 1 shows the general characteristics of interview participants.

**Table 1:** General characteristics of participants.

Characteristics	N (%)
**Gender**	
Male	8 (23.53%)
Female	26 (76.47%)
**Age group**	
22–29	17 (50.0%)
30–39	11 (32.4%)
40–49	6 (17.6%)
**Healthcare professional**	
Physician	12 (35.29)
Pharmacist	12 (35.29)
Nurse	10 (29.42)
**Years of experience in the current position**	
1–5 years	15 (44.1%)
6–10 years	8 (23.5%)
11–15 years	5 (14.7%)
16–20 years	5 (14.7%)
>20 years	1 (3.0%)
**Healthcare organization**	
Jordan University Hospital	20 (58.8%)
King Abdullah University Hospital	14 (41.2%)

### Barriers to optimal vancomycin prescribing and administering practice

The barriers the HCPs perceived concerning the optimal prescribing and TDM practice were mainly related to the following major sub-themes: (1) barriers pertaining to the healthcare professionals; (2) barriers pertaining to the healthcare system and working environment.

### Barriers pertaining to the healthcare professionals

#### Healthcare professionals’ concerns

HCPs concerns that can lead to noncompliance to guideline recommendations regarding vancomycin prescription were categorized into three subthemes:

a) Family’s response to stopping antibiotics and the fear of being blamed as a barrier to optimal vancomycin use in real daily clinical practice.

*"… the clinical picture and other factors control our use of antibiotics*, *not just the guidelines; for example*, *some families are nagging and troublemakers and you (the doctor) are afraid that if you stop the antibiotic and the patient relapses that you would be blamed*, *so if the kidney function is good*, *you would keep the patient on vancomycin*.*"*

(Participant 11—neurosurgery resident).

b) Fear of vancomycin adverse effects

*"In the NICU*, *the problem is that neonatologists don’t usually increase the vancomycin dose to reach a target of 15–20 mg/L even when it is indicated because they are afraid of vancomycin’s toxicity"*

(Participant 12—clinical pharmacist)

c) Fear of potential undiagnosed infections

*"We feel that we overprescribe antibiotics against guideline recommendations*. *For instance*, *we know that the case is a case of drug-induced fever or autoimmune disease*, *but still*, *we give antibiotics because deep inside you will not be comfortable if the patient needs an antibiotic for some reason and you don’t give it*, *so frankly we overprescribe antibiotics (vancomycin)*. *We give fluids and take levels to keep the patients safe"*

(Participant 18—chief internal resident).

As for TDM, the same resident reported that TDM might be overused in the ICU due to concerns related to the critical situation of patients

*"We may perform TDM on a daily basis for ICU patients because they are critical and their kidney function changes continuously*, *so they need adjustment every day* …*we do it even if the level is not valid because serum creatinine changes… we also may tell nurses to delay the next dose until we tell them to give it depending on the level results"*

(Participant 18—chief internal resident).

#### Perceived lack of benefit of TDM

Some HCPs perceived vancomycin TDM as unnecessary, which may lead to noncompliance with TDM guidelines

*"Vancomycin TDM is not really necessary*. *There are more important things to do*… *you would do TDM for a patient on digoxin*, *or patients taking neuro-medicines or antipsychotics*, *for those medications we need to take levels"*

(Participant 28 -internal resident).

#### Negative perception towards prescribing guidelines

Interview findings revealed that residents and clinical pharmacists seemed to be aware of available international guidelines and support resources for antibiotic prescribing in general and vancomycin in specific and were careful to familiarise themselves with all the evidence and knowledge about vancomycin dosing and monitoring. However, they expressed concerns about the simplicity of guidelines and their lack of updated information.

*"We have guidelines*, *but they are not used*, *they are outdated*, *and they don’t include everything"*

(Participant 20 -pediatric resident)

*"If something is straightforward*, *we use guidelines*, *but we don’t see them useful for complicated cases*, *we rely on experience"*

(Participant 7- emergency resident)

Furthermore, another HCP pointed out how prescribers questioned the applicability of evidence from international guidelines to real-world prescribing population, as indicated below:

*"We have international guidelines*, *there are reasons why doctors don’t follow guidelines*, *their recommendations may contradict their experiences and they may consider that the management of diseases in actual practice in Jordan is different from USA or U*.*K*. *because resistance patterns differ"*

(Participant 27- pediatric resident).

### Barriers pertaining to the healthcare system and working environment

#### Lack of guidelines to guide TDM

HCPs perceived the lack of clarity of guidelines in vancomycin TDM as a barrier to optimal practice:

*"Again*, *the barriers would be the fact that we don’t have clear guidelines here… for changing the dose based on the trough level*, *we would depend more on the experience*, *so we might change*, *for example*, *the dose by 10–15% of the original dose depending on the case*. *Actually*, *there is no fixed ratio*."

(Participant 8: internal resident)

#### Prescribing culture

The decision to initiate vancomycin is usually made by consultant physicians, especially in complicated cases. However, vancomycin can also be started by residents in simple cases or during night admissions, and then the decision is confirmed by the consultant.

*"Consultants along with residents prescribe vancomycin …*. *It depends on the situation*. *If I am on a night shift and the case is clear for example*, *bacterial pneumonia*, *it is not reasonable to call the consultant in the middle of the night*, *we start it*, *and the following day during the round*, *we finalize the decision*….*I don’t always agree with consultants regarding their prescription of vancomycin*, *I think there is abuse"*

(Participant 20 -pediatric resident)

Residents expressed their hesitancy to share their concerns regarding vancomycin prescription with consultants and stated that they would just follow the consultant’s recommendation without questioning their judgment, even if they thought that the decision was not correct.

*"Residents are responsible for prescription unless in complicated cases*… *We have a protocol in our NICU unit*. *Some consultants may give vancomycin even when not indicated*. *Vancomycin is frequently prescribed (in about 20% of patients)*. *I am not always convinced with vancomycin prescription decisions*, *we have misused*, *but I don’t share my opinion with consultants*.*"*

(Participant 11: neurosurgery resident)

*"If the consultant insists on prescribing vancomycin*, *we just follow his recommendation even if it doesn’t follow the guideline*, *we don’t question his judgment"*

(Participant 32, internal resident)

#### Lack of effective communication among healthcare providers

Ineffective communication between healthcare providers may result in ineffective dose adjustment, delayed doses, or misinterpreted samples.

*"As pharmacists*, *if we have a recommendation about a dose change*, *we have to communicate with the medical team in charge of the patient*, *you have to communicate with the senior resident or the consultant*, *and sometimes this is difficult*, *and the required recommendation is not implemented"*

(Participant 26 -clinical pharmacist)

*"As nurses*, *we suffer until the junior physicians come to do a scheduled sample; you call them and tell them to come*, *sometimes they come*, *and sometimes they don’t*, *and sometimes they come late*. *So we may do the samples to avoid further delay"*.

(Participant 9—nurse)

*"Sometimes nurses give the dose before we have the chance to take a trough sample*, *you will not find this in records*, *and we wouldn’t know unless we have an odd level*, *for example*, *your patient’s level is 6-7mg/L daily*, *suddenly you would find that the level is 50 mg/L*. *Usually*, *this gives you an indication that the trough sample was withdrawn at an inaccurate time"*

(Participant 10 -internal resident).

#### Lack of support strategies: On-rotation versus out-of-working hours

Prescribers declared that they are better supported during working hours either by the availability of consultants or by requesting consultation on the spot from other colleagues. For example, they can refer to infectious disease specialists and clinical pharmacists who may offer prescribing advice for challenging decisions that are not covered in the local antimicrobial guidelines. However, out of working hours and night shifts, HCPs were less supported and had fewer resources.

*"During consultant rounds in the morning*, *there are infectious disease specialists that we can refer to if there is something not clear or if the patient is not responding; this is helpful*. *However*, *during night shifts*, *such support is not available*, *but we may contact the fellow"*

(Participant 10 -internal resident)

*"During working hours*, *I am in the ward*, *and I provide support regarding appropriate dosing when needed*, *however outside working hours*, *there is no communication; I don’t have to answer my phone outside my working hours they may just give a stat dose at the time"*

(Participant 22 -clinical pharmacist)

#### Work pressure and shortage of staff

Furthermore, for many HCPs, lack of time due to work pressure per se was perceived as a barrier to optimal vancomycin administration. For instance, a nurse revealed that work overload and time pressure would make it easy to make mistakes.

*"I don’t justify*, *it is not a justification*, *but the reality is something else*. *For example*, *as a nurse*, *you have 4*, *or 5*,*6*,*7 patients on antibiotics*. *This number is problematic*, *and work pressure causes stress that generates errors*. *Errors are usually missed information that is important or risky to the patient*.*"*

(Participant 29 -nurse)

The workload was also reported consistently to hinder HCPs from optimal TDM practice.

*"it is difficult to adhere to TDM guidelines in terms of accurate recording of the time of dose administration and sample collection*. *We have a lot of pressure*. *If we were in a private hospital and each of us handled 2–3 patients*, *that would be easy*. *I have to administer medications for 20 patients if we assume that 3–4 patients are on vancomycin it will be difficult to record every dose*, *there is no time and no staff"*

(Participant 14-nurse)

*"Some mix-ups in vancomycin TDM may occur due to work pressure*. *A pharmacist counts the doses of vancomycin once prescribed and marks when vancomycin TDM should be done*, *but they may miss this mark*, *or the pharmacist may not mark the medication sheet if vancomycin was started during the weekend*, *and the TDM may be forgotten*. *It depends on the degree of work pressure in the ward"*

(Participant 17 NICU pharmacist).

#### Lack of institution-wide training opportunities

HCPs seemed to have assumed that, in principle, lack of training was a barrier to optimal TDM performance. Interviews with HCPs revealed that they were keen to participate in training workshops that were assumed to support their needs and fill in the gap in current practice.

*"I don’t remember receiving any training regarding antibiotics; as you know*, *this mainly related to physicians*, *but I remember once we took a lecture about administration*, *I would like more training about antibiotics and TDM*. *I think it will be useful"*

(Participant 30- nurse)

#### Logistics

HCP reported that sometimes logistics might interfere with the efficiency of TDM and the ability to comply with its protocol

*"The problem is that multiple staff is involved in the process of TDM*, *and this confuses things*, *sometimes I can’t do my job because the porter didn’t take the sample to the lab…sometimes this has delayed my work for 4 hours*, *it is not easy to go by the book in actual practice"*

(Participant 1 -clinical pharmacist)

### Potential strategies to optimise vancomycin prescribing and TDM practice

Healthcare professionals were asked to suggest strategies and recommendations for improvement, *to address their needs*. *Their needs were summarized as follows*:

#### Provide training sessions

Overall, there seemed to be a general sense of the need for training sessions for HCP. Most HCPs suggested that providing education and training in vancomycin dosing and monitoring might have the potential to increase their knowledge and confidence during their daily practice.

*"There must be continuous training*, *especially for staff working with pediatrics as their doses are small and not easy to prepare also we see a lot of side effects such as red man syndrome*, *more training would be useful"*

(Participant 3—nurse).

#### Provide a decision support system

HCPs seemed to have assumed that, in principle, the use of decision support systems would be useful and could potentially improve adherence to TDM guidelines to support their needs and fill in the gap in current practice.

*"I think any sort of computerized program is good*, *as far as it has an alarm system that reminds you of the samples that must be drawn for your patient*, *and a calculator where you put patient’s characteristics*, *and it gives you the new dose*, *so it’s more organized and more structured… it will be good* …*"*

(Participant 10 -internal resident)

Moreover, a clinical pharmacist suggested that such a system would be useful for HCPs who are not familiar with doing vancomycin calculations.

*"We took a course about vancomycin calculations*, *but we didn’t complete it*. *We do the calculations based on the equations we took at the university*, *and I don’t feel confident doing them alone*. *If there is a support system*, *it would be useful*.

(Participant 22 -clinical pharmacist)

#### Activating the role of clinical pharmacist

HCPs explicitly referred to the potential role of clinical pharmacists in the vancomycin prescribing process. For instance, a resident spoke from her experience, as indicated below:

*"I feel that it is very useful that we have a clinical pharmacist in NICU*. *She always reviews the medication sheet*, *checks the levels*, *and checks the doses of babies because their weight changes day by day*, *so she follows up on increasing or decreasing doses for each patient and checks the duration of therapy*. *As a resident*, *I am very busy*, *so I can’t catch up on everything they help us*. *Honestly*, *I feel that it is very important to improve practice to have a clinical pharmacist in every ward; this is what I am sure of*. *In NICU*, *their presence is very helpful"*

(Participant 7: pediatric resident)

*"I think clinical pharmacists must be more involved in medication reconstitution*, *especially those that need to be prepared under sterile conditions*, *we are under a lot of pressure*, *and they are more knowledgeable regarding drugs and their interactions*. *I think it would be helpful if they were more involved in drug preparation*, *including vancomycin*. *That would be very helpful to us"*.

(Participant 3 –nurse)

#### Need for national guidelines and protocols

HCPs highlighted the need for local guidelines and protocols that would potentially support their prescribing practice performance.

*"I think it would help if there are local guidelines and protocols based on local antibiograms*. *This would always be helpful to improve practice*.*"*

(Participant 20 -paediatric resident)

An HCP also said that the availability of local guidelines would potentially improve their vancomycin prescribing by reducing variation in vancomycin treatment and management and helping to streamline clinical workflows.

"….*it also standardizes the practice*. *It is very important*, *help to streamline workflow"*

(Participant 18 –chief internal resident)

## Discussion

The present study highlights the complexity of vancomycin prescribing and therapeutic drug monitoring and the need for interprofessional communication, activation of the role of clinical pharmacists, and further education and support to optimize its use.

HCPs were generally not aware of the presence of any guidelines related to vancomycin TDM. Nevertheless, prescribers were aware of the presence of vancomycin prescription guidelines (mainly international). However, they were negatively perceived as outdated and noncomprehensive, and they highlighted the need for guidelines based on local susceptibility patterns. This contrasts with the findings of Chan *et al*. [12], where prescribes found guidelines clear and valuable, which facilitates adaptation of guideline recommendations in their daily practice. In our study, some prescribers reported that they find their patient population different, so it can’t fit the structured guideline recommendations for the average patient. Thus, their prescription may differ. In addition, they doubted the ability of guidelines to guide them in the management of complicated cases. This finding comes in line with the finding of Livorsi *et al*. [17], where nonadherence recommendations were also found to be related to the need for individualized patient care and skepticism towards guideline recommendations. Thus, it is essential for healthcare institutions not only to have evidence-based guidelines but also to ensure that these guidelines are continuously updated and modified to enable them to operate within the local environmental needs.

The major role in vancomycin, dosing, discontinuation, and TDM was reported to be for the consultants. This "etiquette" of vancomycin prescribing and medication management that relies on prescribing autonomy and the principle of non-interference is another dominant barrier in guideline acceptance and renders experience and routine ahead of guidelines when making decisions regarding the prescription. Furthermore, it minimizes the important role of interprofessional communication in patient care, which may compromise patient outcomes [18]. Although the medication management and decision-making process in clinical practice revolves around consultants, a space should be made available for both interprofessional support and autonomous decision-making for juniors to improve practice and reduce errors [19]. An area of possible improvement involves restricting unnecessarily prolonged antibiotic use. Unfortunately, this is a commonly reported practice in various clinical fields due to fear of possible complications if antibiotic treatment is terminated on time [9, 20, 21]. Several centers report that activating the role of a clinical pharmacist improves guideline compliance. For instance, the expansion of pharmacists’ role in an orthopedic unit in Australia ensured correct weight-based dosing and SAP duration [22]. In another recent quasi-experimental study, pharmacists had a pivotal role in optimizing the initial vancomycin doses and dose adjustments [23].

TDM process coordination, especially in wards where nurses were responsible for dose administration and junior physicians were responsible for sample collection, was lacking. Despite the theoretical understanding of HCP of the different TDM steps and the importance of TDM sample timing relative to trough collection for correct dose adjustment decisions, some physicians didn’t take the TDM process seriously due to their belief that it is not detrimental to optimizing patient outcomes. Interprofessional communication seemed also lacking; these communication issues seemed not only to happen outside working hours but also during working hours, as there was a lack of standardized laboratory ordering times and linked post-TDM action. Organizing the TDM process efficiently might be difficult as there is a deficiency in a structured hospital system that coordinates sampling times relative to dosing time. Such deficiencies in TDM were also reported in the TDM of immunosuppressant tacrolimus, where trough levels were drawn appropriately only in 25.9% of the cases. Furthermore, only 38.1% of the drug administrations occurred within one hour of laboratory study collection [24].

Our previous work revealed that in pediatric wards at JUH [10], discrepancies between the recorded and actual times of dose administration were noted in 83.9% of audited occasions. In comparison, such discrepancies were noted in 82.7% of audited times of sample collection. In the present study, HCPs were aware that recorded times were inaccurate. However, they explained that the main driver for this inaccuracy was related to work pressure, as recording accurate times seemed to have a lower priority compared to other daily tasks for which HCPs are responsible.

Several optimization strategies have been suggested, including continuous training and providing local guidelines, which is essential. However, they need to be implemented in addition to other measures, such as reducing workload and activating the role of clinical pharmacists. Additional measures include providing support systems to better perform vancomycin dosing and monitoring. Such a system contains alarms to remind them of scheduled blood sample collection and calculators to help them optimize the dose selection. This can be useful since dose changes in actual practice are empirically based on a slight increase or decrease in dosage regimen. Initial pilot studies evaluating such tools in clinical practice reported enchantments in monitoring processes (reduced number of blood samples and side effects) during the study period. However, long-term effects still need to be evaluated [25]. The main limitation of the present study is the convenience sampling technique, as only HCPs interested in vancomycin prescribing and TDM were included in the study.

## Conclusions

For enhancing vancomycin prescription and TDM, the present study highlights the need for creating effective communication networks between HCP, reducing workload, and adapting local guidelines in clinical practice suitable for implementation in the local context. For TDM specifically, a strong organizational structure for the TDM process is needed to consolidate different TDM aspects and accommodate effective interprofessional communication. This is quite important as TDM is not only unique for vancomycin, and such structure, if proven useful, can be utilized for the TDM of other drugs.

## Supporting information

S1 File Interview guide (translated version). (DOCX)
