# Supplementary material for: Vancomycin prescribing and therapeutic drug monitoring: Challenges of real clinical practice
Source: PLoS One. 2023 May 17;18(5):e0285717. doi: 10.1371/journal.pone.0285717 (PMC10191297; doi:10.1371/journal.pone.0285717)
Supplement: S1 File — (DOCX) [file pone.0285717.s001.docx]

***Vancomycin prescribing and therapeutic drug monitoring: challenges of real clinical practice***

Mariam Hantash Abdel Jalil ^1^, Rima Ηijazeen^1^, Farah Khaled Abu-Mahfouz ^1^, Khawla Abu Hammour^1^, Maria Hasan Matalqah ^2^, Jwan Saleh Khaleel Albadaineh^3^, Shrouq Khaled AlOmoush ^2^, Montaha Al-Iede ^4 5^

^1^ Department of Biopharmaceutics and Clinical Pharmacy, The University of Jordan, Amman, Jordan

^2^ School of Pharmacy Jordan University of Science and Technology, Irbid, Jordan

^3^ School of Pharmacy Mu'tah University, Kerak, Jordan

^4^ Department of Pediatrics, Jordan University Hospital, Amman, Jordan

^5^ School of Medicine, The University of Jordan, Amman, Jordan

*Corresponding author: m.abdeljalil01@ju.edu.jo

**Appendix 1: Interview guide (translated version)**

**Healthcare professional interview guide**

**Number of study participant …………….**

- Project title: Evaluating the suitability of vancomycin prescriptions: qualitative study for perspectives of healthcare professionals.
- Researcher names ……………………………………...:
- Good morning/ Good evening, my name is XXXX I am conducting a research to learn more about vancomycin prescribing and monitoring practices in your workplace. I would also like to know about any concerns, barriers, and systemic challenges that healthcare professionals may face when making decisions when prescribing vancomycin and monitoring its blood levels. I am very interested in your opinions and experiences on this matter. Re-confirm that:
- This information will help us to better support healthcare practitioners' decision-makers.
- All information we collect in this interview will be kept confidential. The interview will take around 30 minutes.

(Check that the consent form is given to the participant and that it is clear and completed before beginning this interview)

- **General information about the research participants**:

1. Age ………. [year]
2. Gender: male female
3. Are you: a physician nurse pharmacist
4. Availability of colleagues at your place of work (specialist/fellow doctor/consultant/resident)
5. How long have you been practicing the profession?
6. How long have you been in your current position?

**Section 1: Decision to Prescribe Vancomycin - Roles and Responsibilities**

**How to prescribe vancomycin**

- Consider that I have no information on how to prescribe vancomycin. Can you explain to me how you prescribe vancomycin in terms of indications, dosages, and how it is administered and monitored?
- How often do you prescribe vancomycin? How confident are you in prescribing vancomycin? How confident are you that you are prescribing vancomycin appropriately each time you write a prescription?
- What makes you feel less confident about prescribing vancomycin?
- Are there complications in prescribing vancomycin?
- In your team, who makes the final decision on prescribing vancomycin? Then who is responsible for writing the prescription? Do you agree with the person making the description decision?
- How are these decisions made outside working hours and during night shifts? At the weekend?
- Who is responsible for giving the dose to the patient? Who is responsible for monitoring TDM vancomycin? Who is responsible for taking blood samples from the patients? Who is responsible for recording the sampling time? Who is responsible for recording the dosing time? Who is responsible for making appropriate changes to the vancomycin dose based on TDM results?
- Is there anyone else involved in the process of prescribing vancomycin?
- When you decide to prescribe vancomycin (you or the doctor in charge), what do you base your decision on?
- Can you tell me what factors that should be considered when prescribing vancomycin? What indications do you consider when prescribing treatment with the antibiotic vancomycin?
- What role do vital signs and physiological indicators play in prescribing vancomycin? What role do microbiological assays play in prescribing vancomycin for related decision-making? What are the most useful microbiological tests? Gram stain, bacteria name, sensitivities...etc.
- Do you think vancomycin is prescribed for the correct period?

**Section II: antibiotic treatment guidelines and local recommendations to support current practices, education and training.**

- When you plan to prescribe an antibiotic such as vancomycin, what kind of references do you use or consider to support your decision? Do you use specific treatment guidelines? Is it specific to your workplace?
- Do you use local or international treatment guidelines for vancomycin?

**If yes….**

- When and how do you get them?
- Is it difficult to access?
- How often do you use them?
- How much do you trust them?
- What is useful from them?
- h. and how can it be improved?

**If your answer is No**

a. If you don't use it, why not?

b. What other resources do you use to support the prescribing decision?

- How often do you use and follow the treatment guidelines for vancomycin?
- Did these treatment guidelines, or did the guideline help you learn to prescribe vancomycin better? If yes, then how?

If not, why?

- Is there adherence to treatment guidelines recommendations?

- What do you think are the factors that influence your adherence to the recommendations in the guidelines?

**Section III: Therapeutic Drug Monitoring (TDM)**

- Do you use the TDM when prescribing vancomycin?
- Is there a system for the pharmacy department to follow up on vancomycin levels and dose changes? Describe it, if any.

If the answer is yes, in which cases is it used?

- What are the guidelines you follow in TDM?
- Who is responsible for monitoring/reviewing blood level results?
- After that, who makes the decision to change the dose?
- Is it difficult to remember to take blood samples at the correct time? If the answer is yes, why?
- Do you have problems knowing the correct sample collection time?
- Is there a specific routine for blood sample collection, or does this depends on the doctor's request?
- Where do you write the time of sampling?
- How can the pharmacist or physician know when the blood sample was collected relative to the last dose so that he/she can know how to calculate the next dose?
- What are the challenges of vancomycin TDM?
- What do you think is the biggest challenge with TDM using the AUC method? (if used)
- What can be done to facilitate vancomycin dosing and monitoring?
- What can the pharmacist do to facilitate the process of determining and monitoring vancomycin doses?

**Section IV: Potential Support in the Future**

- What support activities are available to help you prescribe vancomycin?

a. Why and how to use methods?

b. What do you think about all the support currently available at this hospital, is it enough, or is there room for improvement? Any suggestions?

- What is missing? What kind of support and resources do you and other colleagues need?

- Do you think you would benefit from more education and training?

- Do you have any comments, questions, or suggestions to improve the description of vancomycin and adjust its doses?

- Do you have questions? Is there anything you would like to add?
